# Carbon-Free Conversion of SiO_2_ to Si via Ultra-Rapid Alloy Formation: Toward the Sustainable Fabrication of Nanoporous Si for Lithium-Ion Batteries

**DOI:** 10.1021/acsami.3c02197

**Published:** 2023-07-19

**Authors:** Zhen Fan, Wei-Ren Liu, Lin Sun, Akira Nishio, Robert Szczęsny, Yan-Gu Lin, Shigeto Okada, Duncan H. Gregory

**Affiliations:** † WestCHEM, School of Chemistry, University of Glasgow, Glasgow G12 8QQ, United Kingdom; ‡ Department of Chemical Engineering, Chung Yuan Christian University, R&D Center for Membrane Technology, Research Center for Circular Economy, No. 200, Chun Pei Rd., Chung Li Dist., Taoyuan 32023, Taiwan; § Institute for Materials Chemistry and Engineering, Kyushu University, 6-1, Kasuga-koen, Kasuga 816-8580, Japan; ∥ Faculty of Chemistry, Nicolaus Copernicus University in Toruń, ul. Gagarina 7, 87-100 Toruń, Poland; ⊥ Research Division, National Synchrotron Radiation Research Center, Hsinchu 30076, Taiwan

**Keywords:** Microwave-Induced Metal Plasma (MIMP), SiO_2_ Reduction, Dealloying, Nano Si, Lithium-Ion Batteries (LIBs)

## Abstract

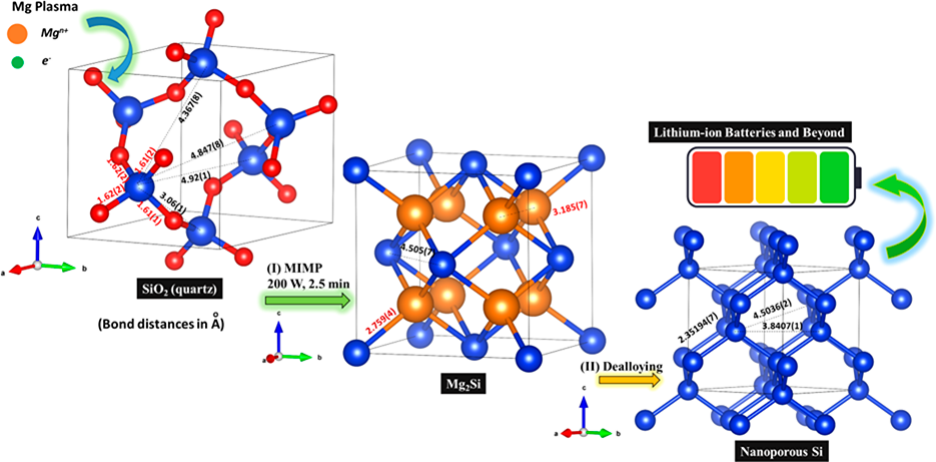

Silicon has the potential to improve lithium-ion battery (LIB) performance substantially by replacing graphite as an anode. The sustainability of such a transformation, however, depends on the source of silicon and the nature of the manufacturing process. Today’s silicon industry still overwhelmingly depends on the energy-intensive, high-temperature carbothermal reduction of silica—a process that adversely impacts the environment. Rather than use conventional thermoreduction alone to break Si–O bonds, we report the efficient conversion of SiO_2_ directly to Mg_2_Si by a microwave-induced Mg plasma within 2.5 min at merely 200 W under vacuum. The underlying mechanism is proposed, wherein electrons with enhanced kinetics function readily as the reductant while the “bombardment” from Mg cations and electrons promotes the fast nucleation of Mg_2_Si. The 3D nanoporous (NP) Si is then fabricated by a facile thermal dealloying step. The resulting hierarchical NP Si anodes deliver stable, extended cycling with excellent rate capability in Li-ion half-cells, with capacities several times greater than graphite. The microwave-induced metal plasma (MIMP) concept can be applied just as efficiently to the synthesis of Mg_2_Si from Si, and the chemistry should be extendable to the reduction of multiple metal(loid) oxides via their respective Mg alloys.

## I Introduction

The growing demand for high energy- and power-density lithium-ion batteries (LIBs) calls for next-generation electrode (especially anode) materials.^1−3^ Silicon has been viewed as one of the most promising anode materials due to its abundance, safety, environmental friendliness, low working potential (∼0.2–0.3 V vs Li/Li^+^), and high specific capacity (∼4200 and 3579 mAh g^–1^ for Li_22_Si_5_ and Li_15_Si_4_, respectively) when compared to the current commercial graphite anode (which has a specific capacity of ∼372 mAh g^–1^ for LiC_6_).^2,4−7^ However, the large volume changes (>400%) that occur on cycling generally lead to the pulverization of particles and rapid capacity decays/failures.^4−8^ The strategy of decreasing the Si particle size to the nanoregime has been well-investigated so as to avoid the pulverization issue, and Si nanoarchitectures have been extensively investigated for Si anodes in LIBs.^2,4−14^ Among the various nanostructures, nanoporous (NP) Si engineered from Si alloys (e.g., Al–, Ca–, Li–, and Mg–Si alloys, etc.) using facile scalable dealloying methods has demonstrated high performance and stability.^2,15−21^ Notably, the Mg_2_Si alloy has been widely studied due to its low capital cost, mild dealloying conditions, and the outstanding performance of the as-obtained NP Si products.^2,22−24^ Various dealloying methods of Mg_2_Si disclosed to date include air-oxidation (or thermal dealloying, forming MgO and NP Si), nitridation (generating Mg_3_N_2_ and NP Si), dealloying in molten metal/salts (yielding Mg_3_Bi_2_/Mg_2_Sn and NP Si), vacuum dealloying (forming Mg vapor and NP Si), and metathesis reactions (e.g., with ZnCl_2_, SiO_2_, SiO, etc., toward NP Si and MgCl_2_, Zn, MgO, etc.).^2,15,16,22−29^ However, the Mg_2_Si precursors that have been used were either purchased as commercial products or synthesized by high-temperature long-duration methods from Mg–Si elemental mixtures.^2,15,16,22,25−29^

**Scheme 1:**
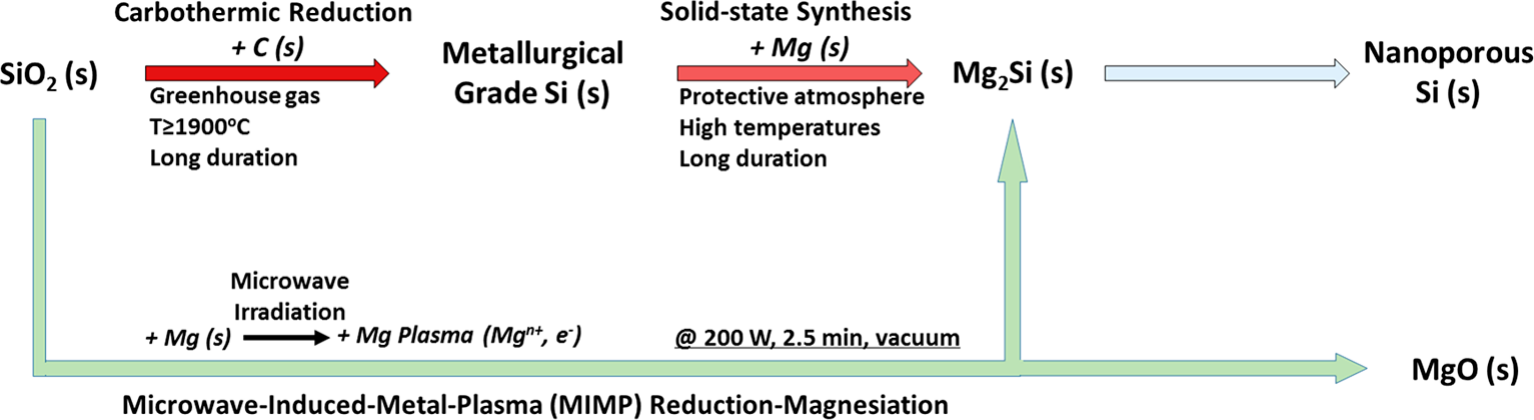
MIMP Method Used in Alternative SiO_2_ to NP Si Routes

The very stable Si–O bond with a high dissociation energy of 460 kJ mol^–1^ makes conventional fabrication of Si from its raw feedstocks energy-intensive.^30−32^ Today’s SiO_2_-to-Si industry still overwhelmingly depends on carbothermal reduction (a 19th century chemical innovation), which is generally performed at temperatures over 1900 °C using electric arc furnaces, thus consuming large amounts of electrical energy and posing serious environmental costs (e.g., greenhouse gas emissions).^33−38^ Correspondingly, the sustainability benefit of nano Si products for LIBs is all but negated by the energy and environmental demands from processing, almost by analogy to Si photovoltaics.^30,33,39^ Routes to prepare Mg_2_Si and NP Si from SiO_2_ that can remove the requirement for carbothermal reduction are extremely attractive alternatives. By employing natural mineral feedstocks or by recycling the vast supplies of glass/quartz waste, it becomes possible to engage in the routine sustainable manufacture of silicon, both for LIBs and for other valuable technologies (Scheme 1).^40^ Magnesothermal reduction, a common process in metallurgy, was proposed to displace O from SiO_2_ using Mg vapor at 500–900 °C in order to produce solid MgO and Si.^40^ Recently, the magnesothermal reduction of SiO_2_ to Mg_2_Si was also reported.^41^ However, these reactions are difficult to practically achieve due to a lack of stoichiometric control and the complex kinetics/thermodynamics of Mg–SiO_2_ reactions. Consequently, pure products are not reproducibly achieved (due to formation of silicate byproducts etc.), leading to low yields (typically <50%) and poor repeatability.^40,42−45^ Interestingly, by conducting magnesothermal reduction under a partial vacuum of ca. 1.4 × 10^–1^ torr, separating the Mg and SiO_2_ starting materials, and using a carrier Ar gas to guide the Mg vapor to the solid SiO_2_ powders, Yoo et al.^45^ reported a reduced reaction duration and improved Si product purity. Their powder X-ray diffraction (PXRD) results illustrated that Mg_2_Si formed initially (via rapid vapor transport), which then reacted with SiO_2_ to form nano Si. However, the experiment required high temperatures (675 °C) and the setup was complicated.^45^ The electrification of mining and materials processes is a long-term societal goal, and the fabrication of Si nanomaterials for LIBs from the electrochemical deoxygenation of silica is an attractive prospect.^30,40,46,47^ Nonetheless, elevated temperatures (e.g., >850 °C), specific molten salts (e.g., CaCl_2_), additional materials (e.g., cathode additives), and protective atmospheres (often an Ar flux) have proven mandatory in these electrochemical processes, leading to energy-intensive production, demanding equipment requirements, low yields, and low product purity.^30,40,46,47^

Herein, we disclose a rapid and efficient chemical SiO_2_ reduction process with no inherent infrastructure requirements (Scheme 1). A metastable Mg plasma was adopted to achieve the ultrafast reduction-magnesiation of SiO_2_ to Mg_2_Si within 2.5 min by microwave (MW) irradiation at merely 200 W (at 2.45 GHz) under a vacuum at lab scale. Unlike conventional, thermally driven bond-breaking processes, free mobile electrons act readily as the reductant with solid SiO_2_. Simultaneously, the constant “bombardment” from Mg cations and electrons promotes the spontaneous and ultrafast nucleation of solid Mg_2_Si and MgO. Then, via a simple thermal dealloying in air, magnesium is oxidized, and the silicide is converted to high surface area 3D nano Si with ultrafine ligaments and multiscale porosity. Li-ion cells incorporating the resulting nano Si as anodes (even without further treatment, the formation of composites, or by electrolyte optimization with additives) demonstrated remarkable cycling stability, high capacity, and exceptional rate capability. Very similar results can also be obtained via the same synthesis method when SiO_2_ is replaced with Si as the source of silicon. This study reveals how the emerging physiochemical concept of microwave-induced metal plasma (MIMP) reaction chemistry can be used to convert SiO_2_ (or Si) from either natural feedstocks or recycled waste to useful material commodities such as Mg_2_Si and especially nanostructured Si, whether for LIBs or ultimately other technologically important applications.

## II Results and Discussion

### MIMP Reduction-Magnesiation of SiO_2_ to Mg_2_Si

**Figure 1:**
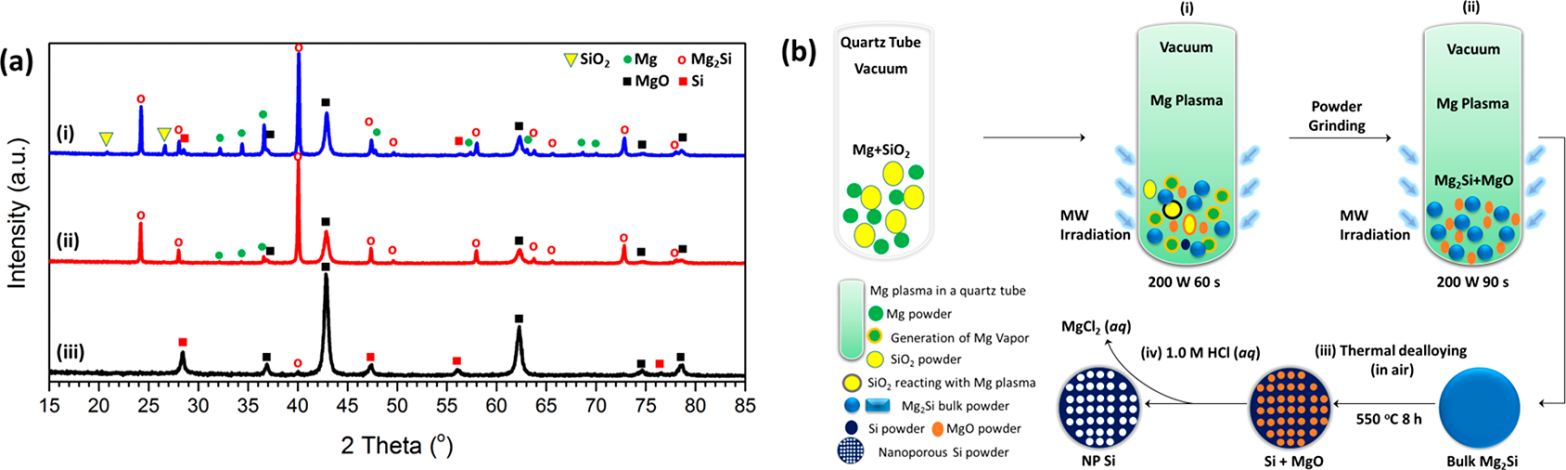
(a) *Ex situ* PXRD results of samples at different synthesis steps: (i) after the first MW irradiation of 60 s, (ii) after the second MW irradiation of 90 s, and (iii) after the thermal dealloying under air atmosphere at 550 °C for 8 h. (b) Schematic of the MIMP-assisted synthesis of NP Si from SiO_2_.

Previously, we devised the MIMP method for the synthesis of intermetallic Mg_2_Sn, demonstrating that irradiating a fine Mg powder in a microwave field generates Mg plasma within seconds, capitalizing on the volatility of Mg and the easy loss of outer-orbital electrons from Mg atoms in the gas phase.^48,49^ Herein, we show that microwave-induced Mg plasma can effectively reduce SiO_2_ (quartz)—despite the fact that SiO_2_ itself is MW-transparent^48^ —to form Mg_2_Si within 2.5 min (see Figures 1a(i),(ii) and S1; for full details, see the Experimental Section and Supporting Information 1).

To achieve this reduction, a mixture of Mg and SiO_2_ powders was irradiated by MWs at 200 W under a static vacuum of 1.0 × 10^–1^ mbar in a single-mode cavity MW reactor. The process consistently generated a green plasma that was composed of positively charged Mg cations, metastable neutral particles/clusters, and electrons (e^–^) (Supporting Videos 1 and 2). This phenomenon occurred within a few seconds (<10 s) of MW irradiation. A very similar phenomenon was consistently observed in our previous study of MIMP reactions using Mg and Sn reactants.^48^ The *ex situ* PXRD patterns show that the majority of SiO_2_ was transformed to Mg_2_Si (as the major crystalline phase) in the first MW irradiation period of 60 s (with the expected generation of MgO byproduct).^50−52^ The flat background in Figure 1a(i) would also indicate the absence of any obvious amorphous phases.^40^ An almost negligible amount of Si was detected by PXRD (Figure 1a(i),b(i)),^53^ suggesting that any Si formed via reduction is almost spontaneously magnesiated by the Mg plasma in the vicinity of the powder mixture. Thermodynamically, the products of conventional magnesothermal reductions of SiO_2_ are sensitive to Mg; the SiO_2_ molecules ratio in a predictable way, where higher ratios (≥2:1) lead preferentially to the formation of Mg_2_Si over the reduction to Si (eqs 1 and 2) .^40−45^
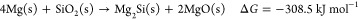
1
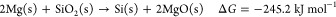
2

The powder mixture was ground and irradiated by MWs for a second cycle at the same input power and vacuum conditions for 90 s; all of the SiO_2_ and the residual Si reacted with the Mg plasma (generated from the Mg reactant powder that remained from the first irradiation, Figure 1b) to yield Mg_2_Si and MgO (alongside a tiny quantity of unspent Mg, Figure 1a(ii)).^50−54^ The fact that the residual Si is so readily removed in the second irradiation is perhaps not surprising, given the propensity for Si as a semiconductor to itself interact with the microwave field. Perhaps more surprising is the ease with which SiO_2_ (itself electrically insulating and MW-transparent with a low dielectric loss; loss tangent, tan δ ≈ 2.3 × 10^–7^)^55^ is consumed in the MIMP reaction; this is a testament to the reactivity of Mg in the plasma state.

**Figure 2:**
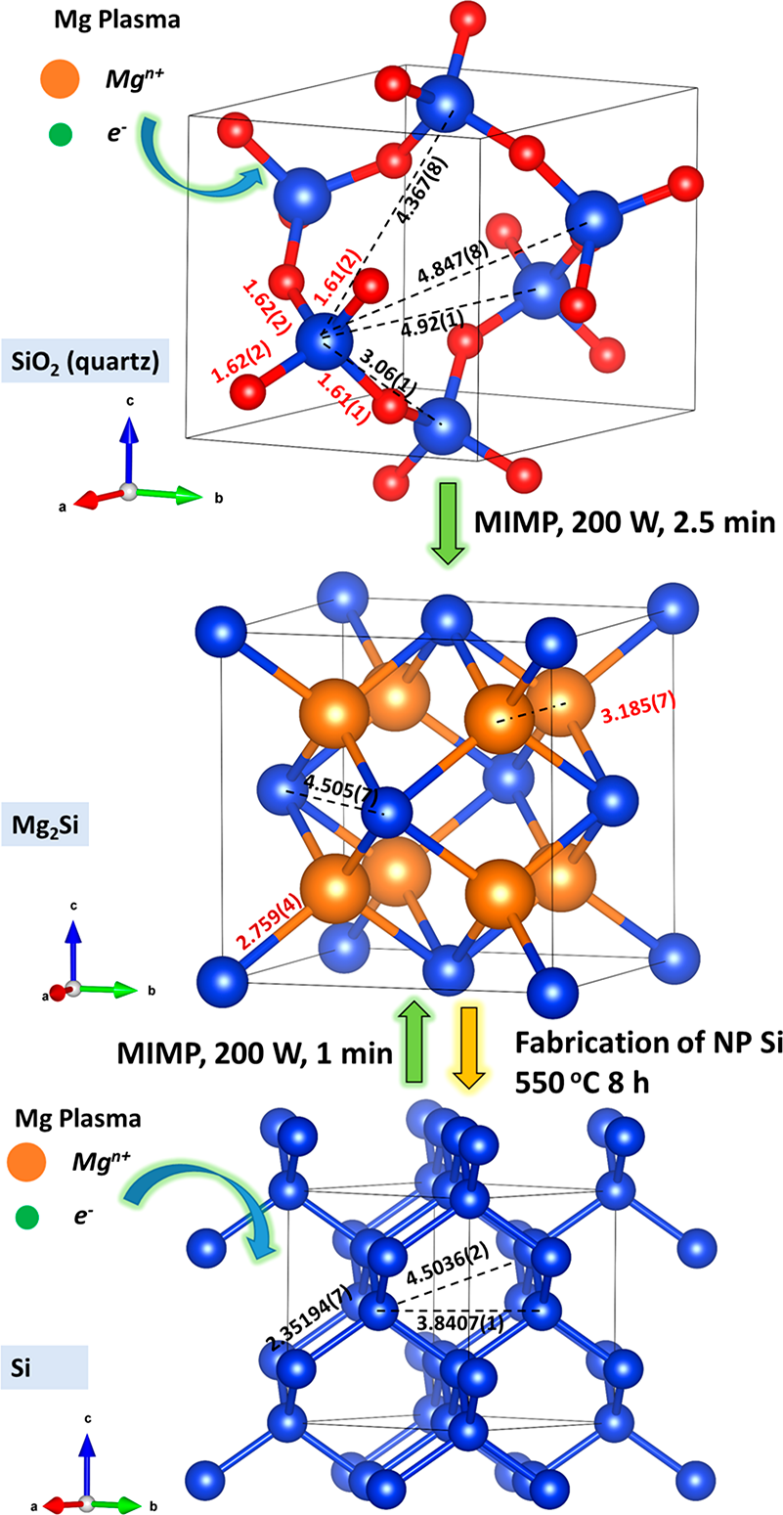
Crystal structure evolutions during the MIMP and thermal dealloying reactions: from top to bottom, quartz (structure from ref (50)), Mg_2_Si (structure from ref (51)), and Si (structure as refined against PXRD data for NP Si). Atoms: red for oxygen, royal blue for silicon, orange for magnesium. Bond distances are given in Å.

Compared to the conventional carbothermal reduction of SiO_2_ to Si, which demands time and considerable thermal energy in order to drive the bond-breaking chemistry of SiO_2_, our method requires a brief, low energy input, emits no CO (or CO_2_) gas, and is complete within 2.5 min (Figure 2).^33,48^ Our MIMP method benefits in two ways from the kinetics of the plasma-related interactions in the high-frequency electromagnetic field of 2.45 GHz. (1) The Mg cations in the plasma phase have already lost the valence (and potentially other outer-orbital) electrons and, while themselves may not be reductive, provide the “free” electrons in the plasma phase to act as the reductant required to break the Si–O bonds. This is broadly analogous to electrochemical reduction/catalysis reactions, which employ electrons as catalysts or reductants.^30,48,56^ In the MIMP case, the kinetics of the plasma phase can also be significantly enhanced by the high-frequency MW field. (2) The rapid “bombardment” of Mg cations (and neutral particles/clusters) and electrons with Si and O species in the high-frequency electromagnetic field may intrinsically promote the nucleation of the thermodynamically stable Mg_2_Si and MgO solids when the metastable plasma reacts with SiO_2_. The relatively small mass and size of Mg cations (the ionic radius for Mg^2+^ is 0.72 Å) and of electrons facilitate their rapid motion in the MW field with a minimal power input.^57−59^ The reaction of SiO_2_ to Mg_2_Si (a cubic antifluorite structure, as opposed to Si, which is cubic diamond) is governed by the reaction stoichiometry, the vacuum conditions (as reported to favor Mg vapor formation and transportation, and, in our case, favor the formation of Mg plasma),^45^ and the Mg plasma-rich environment during the MIMP reaction. In the first two respects, the reaction is ostensibly no different from the conventional magnesothermal formation of Mg_2_Si from SiO_2_ with excess Mg.^40−44^ Additionally, the relatively open structure of SiO_2_ (quartz), which supports large Si–Si distances, may expedite the diffusion of plasma ions/electrons (Figures 2 and S7). Overall, the reaction could be represented as shown in eq 3, which highlights the role of the reactive Mg plasma:^30,33,60^
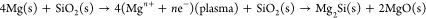
3

In principle, and given the diffraction evidence in Figure 1a, it is feasible that the reaction proceeds via an initial magnesothermal reduction step to Si, followed by a virtually instantaneous magnesiation to Mg_2_Si. It is technically extremely challenging to obtain the temporally and spatially resolved direct experimental evidence that would allow a model reaction mechanism to be developed.^48,61^ However, *in situ* characterization techniques such as powder neutron diffraction and time-resolved spectroscopy are likely to be able to probe the MIMP reaction process in more detail. Interestingly, further experimental results demonstrate that single phase Mg_2_Si can be synthesized via the MIMP method from bulk Si powders within 1 min (Figures 2 and S2, the Experimental Section, and Supporting Information 1). So, reduction-magnesiation from silica is not the only MIMP route to magnesium silicide (and formation of MgO is not a vital part of the process).

### Nanoporous (NP) Si

**Figure 3:**
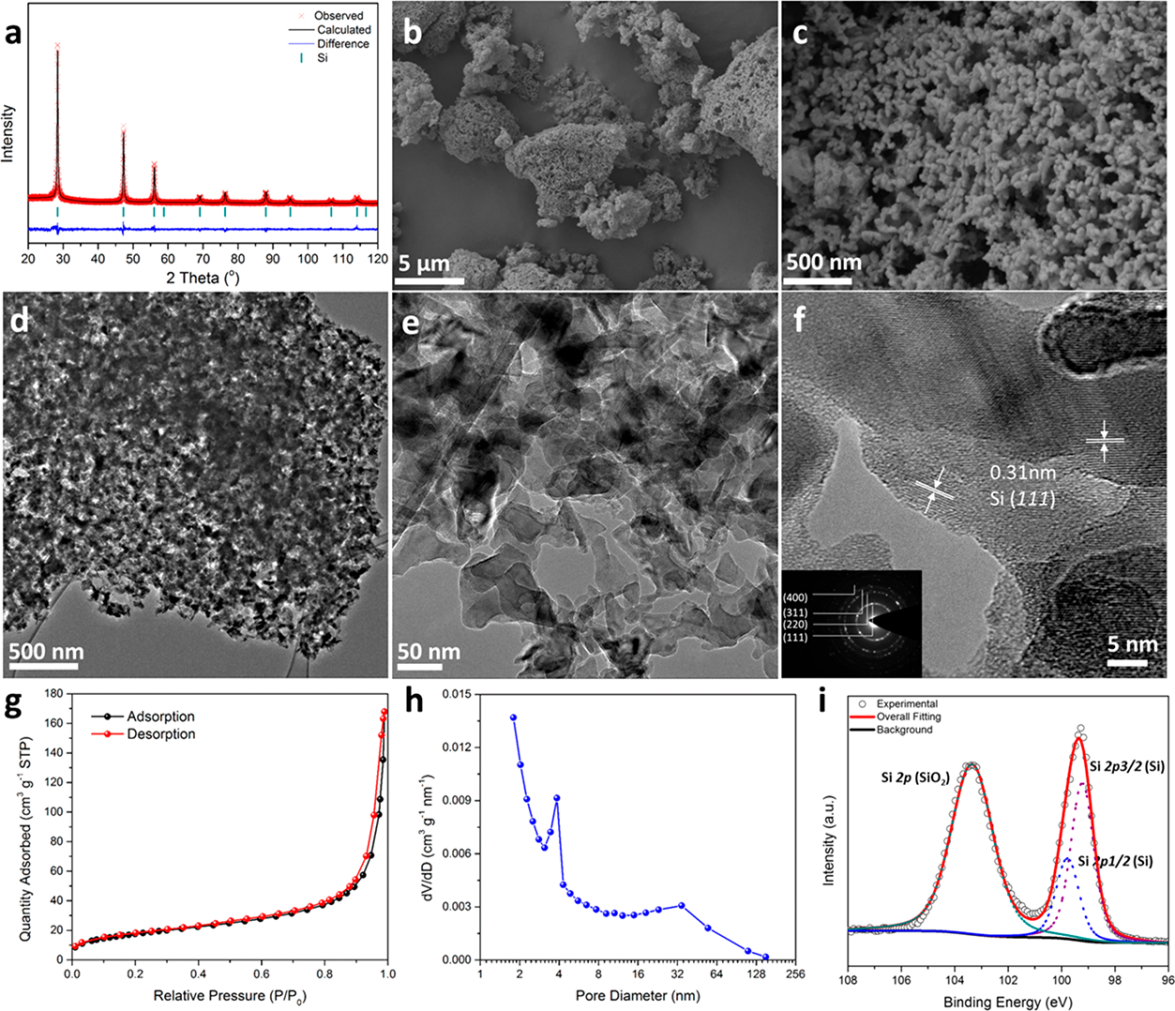
Characterization of NP Si. (a) Profile plot from the Rietveld refinement against experimental PXRD data; (b) low- and (c) high-magnification SEM images; (d) low-, (e) medium-, and (f) high-magnification TEM images, respectively (inset in (f) is the SAED pattern for the area in (f), see also Figure S4); (g) BET N_2_ physisorption curves; (h) the pore size distribution curve obtained by applying the Barrett–Joyner–Halenda (BJH) desorption method; and (i) Si 2p region of the XPS spectrum.

Mg_2_Si is well established as a suitable precursor for the synthesis of NP Si, and various routes have been developed to fabricate NP Si from the silicide for use as an anode for LIBs.^2,15,16,22−29^ Herein, we dealloyed Mg_2_Si via simple thermal oxidation at 550 °C for 8 h in air (Figures 1b(iii) and 2).^26^ During this process, the Mg in Mg_2_Si is oxidized to MgO, which then phase separates to leave a porous structure of Si as the final product.^26^ The resulting PXRD pattern (Figure 1a(iii)) affirms the coexistence of Si and MgO with a trace amount of residual Mg_2_Si. Subsequent washing and drying (Figure 1b(iv)) removes all MgO (including any byproduct from the original Mg_2_Si synthesis) and remnant silicide to yield phase-pure, crystalline Si. Rietveld refinement against experimental PXRD results confirms the cubic diamond-C structure (space group *Fd*3*m*, no. 227) with a lattice parameter of *a* = 5.4339(8) Å (Figure 3a and Tables S1 and S2). Low-magnification scanning electron microscopy (SEM) images show micrometer-sized agglomerates from a few to tens of micrometers across, which themselves are highly porous (Figures 3b and S3). High-magnification SEM images show that the micrometer-sized NP Si agglomerates are composed of uniform and ultrafine nanoligaments ∼20–95 nm in width, with the majority measuring between ∼20–65 nm (Figure 3c). Gas adsorption measurements revealed that pores were distributed over several length scales (see below), but only those in the region of tens to hundreds of nanometers could be imaged by SEM (Figure 3c). Energy-dispersive X-ray spectroscopy (EDS) spectra and corresponding elemental mapping results exclude the presence of residual Mg (e.g., from Mg_2_Si or MgO) and suggest the uniform distribution of Si across the sample surface (Figure S3).

Low-magnification transmission electron microscopy (TEM) images further confirm that the uniform NP structure is built from fine nanoligaments and nanopores (Figure 3d). The magnified TEM image in Figure 3e shows that the nanoligaments that range from a few to ∼50 nm across are interconnected and that nanopores from a few to ∼65 nm in width are continuous, together forming a 3D NP structure. Transmission X-ray microscopy (TXM) directly reinforces this representation of the 3D microstructure (Supporting Information 2). High-resolution TEM (HRTEM) images reveal that the NP Si product is highly crystalline at the nanoscale, showing measurable lattice distances of 0.31 nm, corresponding to the *d*-spacing characteristic of Si (111) lattice planes (Figures 3f and S5). The selected area electron diffraction (SAED) patterns can be exclusively ascribed to polycrystalline Si and are consistent with measurements taken from HRTEM images (see Figures 3f (inset) and S4).

Nitrogen adsorption isotherms yield type II behavior with characteristics of H3 hysteresis, suggesting that the dealloyed Si material exhibits a wide distribution of pore sizes (Figure 3g).^62^ The measured surface area based on the Brunauer–Emmett–Teller (BET) method was 63.21 m^2^ g^–1^. Notably, the Barret–Joyner–Halenda (BJH) analysis of desorption data indicates a micro-meso-macro pore size distribution (Figure 3h), where values range from <2–130 nm with maxima at ∼2, 4, and 32 nm. Based on the results of the BJH desorption measurements, the porosity was calculated to be 37.97% (Table S3 and Supporting Information 1). The Si 2p X-ray photoelectron spectroscopy (XPS) spectrum in Figure 3i identifies Si peaks at 99.31 and 100.04 eV and identifies the existence of a peak corresponding to SiO_2_ at 103.38 eV (Table S4).^26^ The latter peak is highly likely to originate from surface oxidation of the sample during handling and loading into the XPS chamber. Such oxygen species are also suggested by EDS analysis (Figure S3) and the amorphous spots in the HRTEM image (Figure 3f). These findings are consistent with the ease of oxidation at the surface of Si nanomaterials that has been previously reported.^26,41^ It is valuable to highlight that the sharp peaks and flat background seen in the PXRD diffractograms (even after extended air exposure; see Figure 3a) and the extensive lattice fringes seen in the HRTEM images (Figures 3f and S5) imply that oxidation does not extend to the bulk of the nanoporous Si.

### Electrochemical Behavior

Thin pore walls, large surface areas, and multiscale nanopores have been proposed to enhance the access of an electrolyte to LIB anodes. Such anode structures promote the diffusion of Li^+^ (in nanoligaments), accommodate the volume changes of (de)alloying, and alleviate stresses in the solid phase(s).^2,15,16,22−29^ With these aspects in mind, we evaluated the electrochemical performance of our NP Si. We intentionally made no further optimization to the anode or electrolyte in our test half-cells (e.g., via carbon coating of the NP Si or by incorporation of electrolyte additives, as is often reported^2,25,27,63^) so that we could specifically evaluate the efficacy of the Si material itself.

**Figure 4:**
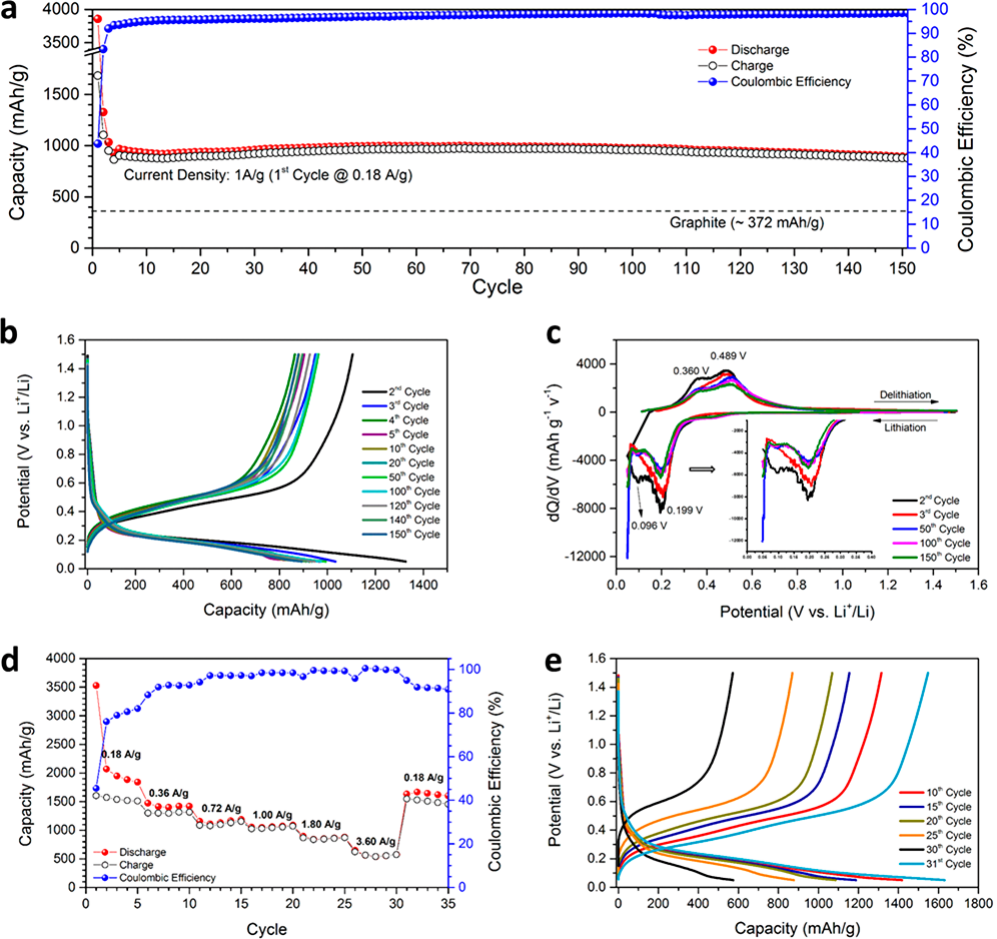
Electrochemical performance of NP Si in half-cells of LIBs. (a) Cycling performance and corresponding Coulombic efficiency at 1 A g^–1^ for 150 cycles (following an initial activation cycle at 0.18 A g^–1^). (b) Galvanostatic curves taken from selected cycles corresponding to (a). (c) Differential capacity plots against cell potential corresponding to (b) (inset: the zoomed-in d*Q*/d*V* curves of the lithiation processes at 0–0.4 V vs Li^+^/Li). (d) Rate performance at different current densities. (e) (Dis)charge potential vs capacity curves corresponding to (d).

Figure 4a shows the (dis)charge capacities and Coulombic efficiencies (CEs) from half-cells cycling between 0.05 and 1.50 V vs Li^+^/Li. The first cycle was activated at 0.18 A g^–1^, delivering discharge and charge capacities of 3858 and 1685 mA h g^–1^, respectively. This large initial irreversible capacity loss is attributed to solid-electrolyte interface (SEI) formation and surface oxidation of NP Si (as indicated to be likely from the EDS/XPS results). Despite many advantages associated with an enhanced rate, the large surface area of active materials has been reported to encourage the loss of Li^+^ to SEI formation and is associated with the poor initial CE of nanostructured Si anodes.^2,11,40^ Moreover, previous studies have shown extensive disruption/formation of SEIs for Si-based alloying anodes with repetitive large-scale electrode expansion and contraction.^2,4−8,11,15,27,64−66^ Naturally, such effects would normally be mediated by surface carbon coating of NP Si and by the use of electrolyte additives (such as fluoroethylene carbonate (FEC) and vinylene carbonate (VC)) to form a thin, stable SEI, as is extensively reported in the literature.^2,4−8,11,15,27,63^ Nevertheless, subsequent cycles at the relatively high current density of 1.0 A g^–1^ showed elevated capacity and excellent stability for the MIMP-synthesized NP Si; discharge and charge capacities stabilized at cycle 4, registering values of 926 and 865 mA h g^–1^, respectively. From cycle 13, the capacities steadily recovered further until, by cycle 69, discharge and charge capacities of 996 and 972 mA h g^–1^, respectively, were obtained with a corresponding consistent increase in CE. Here, the capacity increase in the first few tens of cycles likely originates from a gradual morphological-change-induced activation, as reported in a number of previous studies.^5,6,64,65^ Such structural changes in active materials have been discovered to contribute to the reduction of Li^+^ diffusion resistance at high current densities.^6,64,65^ The capacities showed a slight decrease thereafter, which can probably be attributed to the aforementioned non-ideal SEI, and potential microstructural aggregation from cycle to cycle may also affect the cyclability (see also Postcycling Characterization).^2,4−8,64,66^ Nevertheless, cycles 1–146 consistently delivered an impressive discharge capacity >900 mA h g^–1^ and, after 151 cycles, the half-cell still maintained high discharge and charge capacities of 891 and 877 mA h g^–1^, respectively, both of which are more than twice the theoretical capacity of graphite.^1,2^ Given also that the CE does not drop below 98.0% from cycle 70, the results testify to the intrinsic stability of the NP Si on cycling. By way of comparison, the performance of our NP Si contrasts significantly with that of commercial bulk Si (<10 mA h g^–1^ within 20 cycles at a current of 0.36 A g^–1^) and of typical nanocrystalline Si powders in LIB half-cells (<275 mA h g^–1^ within 100 cycles at a current of 0.2 A g^–1^).^26,27^ In fact, given the absence of additives and lack of cell optimization at the present time, our NP Si compares very favorably with the capacity and stability of many of the nanoporous and/or nanocomposite Si/Si–C materials that have been extensively reported in the literature.^5,15,40,42,43^

Further evidence of the electrode’s stability is provided by the galvanostatic potential curves and the differential capacity against potential (d*Q*/d*V*) plots (Figure 4b,c). The former data show that the main discharge and charge capacities were located at 0.40–0.05 and 0.10–0.60 V vs Li^+^/Li, respectively, confirming the low working potential of NP Si as an anode material, while the latter show how the redox mechanism reaches consistency on extended cycling. Cycle 2 exhibits two cathodic lithiation peaks at 0.199 and 0.096 V (forming Li_*x*_Si)^2^ and two wide delithiation oxidation peaks at 0.360 and 0.489 V vs Li^+^/Li. Cycle 3 shows a broad lithiation peak at 0.199 V, but there is an absence of a lithiation peak at 0.096 V together with a further contribution at <0.07 V (as shown in the inset in Figure 4c), which originates from likely SEI formation and gradual activation and microstructural changes during the (de)alloying of Si and Li over the opening cycles.^2,25,27^ However, in later cycles (cycles 50, 100, and 150 in Figure 4c) there is close overlap of the profiles, indicating increased cycling stability, and the presence of a capacity contribution at <0.07 V after cycle 2 can very likely be attributed to the periodic evolution of the NP Si structure and the alloying/lithiation at low potentials, as mentioned above.^2,5,6,64,65^ The d*Q*/d*V* results thus provide strong evidence for a stable, multistep (de)alloying mechanism for the NP Si anode on (de)lithiation.^25,27^

Figure 4d shows the rate performance of our NP Si product. The cell experienced capacity fading during the first five cycles at 0.18 A g^–1^, which, as described above, can be related to the formation of an SEI (with large volume changes). The cell delivered consistently good capabilities at different current densities over subsequent cycles, delivering average discharge capacities of 1424, 1149, 1064, 865, 576, and 1632 mA h g^–1^ at 0.36, 0.72, 1.00, 1.80, 3.60, and 0.18 A g^–1^, respectively. Corresponding CEs of 92.5, 97.2, 98.5, 99.4, 100.0, and 92.1% were obtained at these rates. The latter drop of CE from cycle 31 as the current density was increased once more to 0.18 A g^–1^ is unsurprising, given that higher electrode volume expansion occurs (as expected) as the capacity increases. Surface modification of the NP Si and the inclusion of electrolyte additives (both contributing to SEI formation) would be expected to mitigate these CE losses, as has been reported in the literature.^2,63^Figure 4e indicates the smooth potential curves that are obtained from cycling at different current densities, demonstrating the stability of the NP Si electrode and illustrating its suitability for high rate (dis)charging. Notably, the working potentials remained within ranges of 0.40–0.05 and 0.10–0.70 V vs Li^+^/Li at high current densities, without any obvious differences from cycles performed at lower rates, demonstrating no serious kinetic limitations.

### Postcycling Characterization

**Figure 5:**
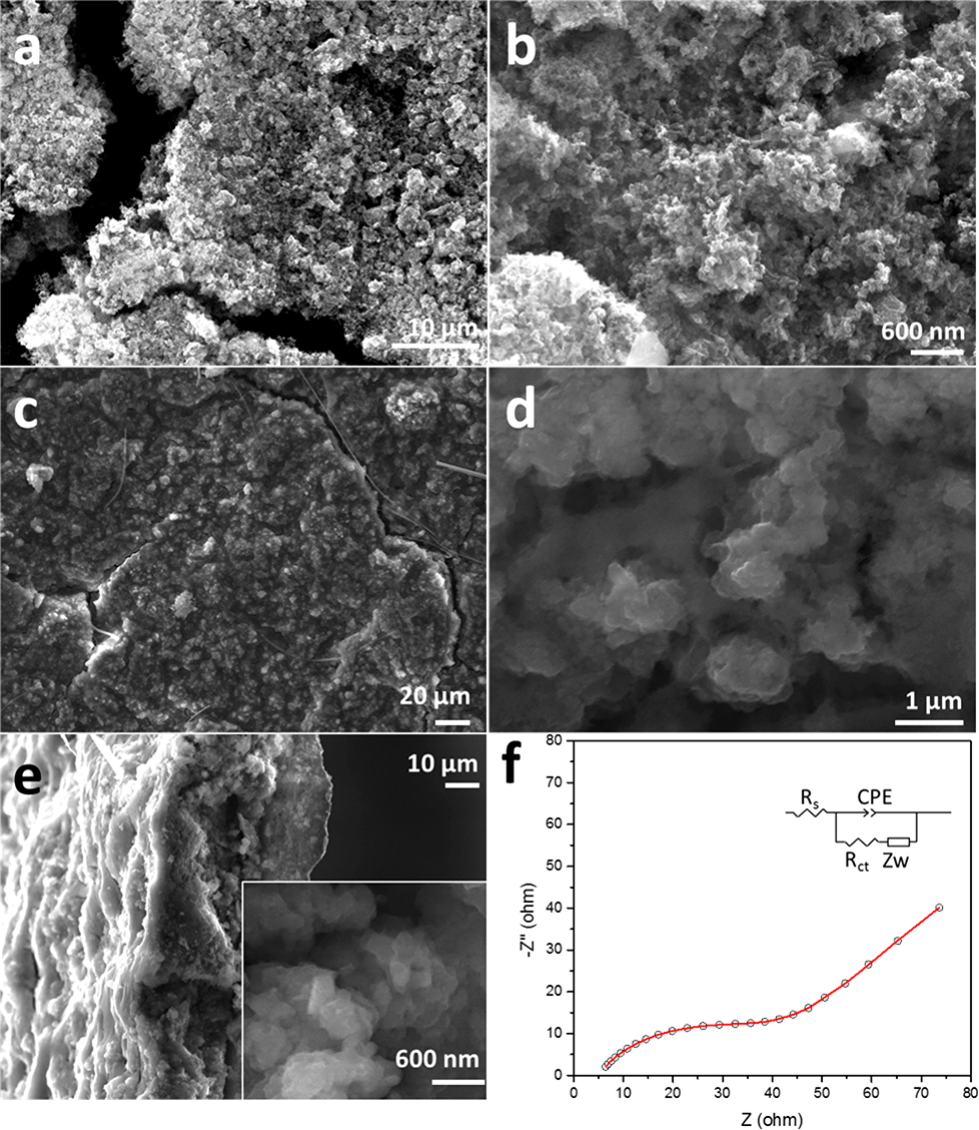
(a and b) SEM images of a fresh NP Si electrode precycling. (c–e) SEM images of the cycled NP Si electrode after 151 cycles: (c) and (d) show surfaces while (e) shows cross sections (inset: a high-magnification SEM image). (f) EIS spectrum of the half-cell after 151 cycles (at an open-circuit voltage). The equivalent circuit is shown in the inset, and the fit to the circuit is indicated by the solid red line.

Compared to micrographs of the as-prepared electrode prior to testing (Figures 5a,b and S6), postcycling SEM images of the NP Si electrode (after 151 cycles; see Figure 5c–e) show that, despite the formation of the SEI, the surface of the electrode remained flat, integrated, and mechanically robust. This confirms that the NP structure successfully avoided the fracturing phenomena experienced by many other Si active materials.^2,27^Figure 5d shows that the ligaments and pores of our NP Si were retained postcycling, although the coarsening of the ligaments and the dilation of some pores is notable. Such an evolution in microstructure is not altogether surprising when considering the continuous volume expansion/contraction and (re)formation of the SEI during (de)lithiation. Cross-sectional SEM images in Figure 5e show that the characteristic NP structure was a consistent feature across the surface and thickness of the cycled electrode. Electrochemical impedance spectroscopy (EIS) measurements taken from the cell after 151 cycles (Figure 5f) demonstrate that values for the surface impedance, charge transfer resistance, and resistance for bulk diffusion (Table S5) are low compared to examples of previously reported Si anodes in LIBs.^2,25,27^

### Outlook

Considering that, thus far, MIMP-synthesized NP Si has been employed without any further optimization (such as surface treatment or electrolyte modification), there remains abundant scope for the NP Si anode to be improved further.^2,25,27,63^ Moreover, clearly, the present study has been restricted to typical lab-scale quantities of material, befitting preliminary coin-cell tests. Further extended full-cell measurements with coin- and pouch-type configurations would be necessary to evaluate the genuine utility and viability of the process and material toward larger-scale applications. Nevertheless, the MIMP method presents credible routes toward the sustainable fabrication of NP Si from SiO_2_ (from either naturally occurring or waste sources) or equally from bulk (waste) Si itself. Assuming that electricity is supplied sustainably from renewable resources, the MIMP process can be completely carbon-free with no greenhouse gas emissions. The effective coupling of fine Mg powders with MWs and the low ionization energies (IEs) of Mg (737.75 and 1450.68 kJ mol^–1^ for the first and second IE, respectively) circumvent issues associated with normally MW-transparent SiO_2_ (provided that an intimate mixture of fine powder is used) to enable direct synthesis to Mg_2_Si.^67^ In fact, although dealloying of the silicide is required for NP Si formation, a more general route toward the fabrication of Si from SiO_2_ could be achieved solely by microwave methods. Si can be synthesized conventionally by solid state metathesis (SSM) between Mg_2_Si and SiO_2_.^68^ Hence, a crude MIMP-synthesized Mg_2_Si/MgO mixture could be coupled successfully with SiO_2_ and Si could be synthesized from SiO_2_ in two steps (reaction 4):
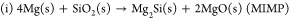

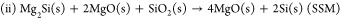
4

The Mg_2_Si + SiO_2_ reaction is exothermic and can self-propagate simply upon activation (by MWs or by other means). Since both the MIMP and SSM processes require relatively modest energy input, the reaction scheme offers an attractive rapid and sustainable production route to Si (and Mg_2_Si, if desired) from SiO_2_. Successfully scaling up the MIMP process will be dependent on surmounting electrical and process engineering challenges as much as overcoming issues associated with chemistry. Working with larger sample volumes will require finding solutions to microwave penetration depth (skin depth) problems, especially for high dielectric loss materials.^61^ One solution is to reduce the MW frequency (since penetration depth scales with wavelength), optimized to balance with the power the material absorbs (proportional to the frequency). Another method is to minimize sample volumes by constructing continuous flow reactor systems. Encouragingly, given the wide access to various types of (bespoke) MW cavities when considering potential flow-synthesis designs, there is reason to be optimistic that the MIMP approach could be successfully scaled. We are evaluating the potential of these alternatives and exploring routes toward scale up with outcomes that will be reported in due course.

## III Conclusions

In summary, a sustainable, ultrafast MIMP route to Si from SiO_2_ through Mg_2_Si via reduction-magnesiation chemistry has been developed. The underlying physiochemical principles can potentially be extended to a tranche of p-block and/or transition metal(loid) (M) oxides whose M–O bonds exhibit dissociation energies no higher than those of the Si–O bond. The MIMP reaction can equally be performed to synthesize Mg_2_Si directly from bulk Si and, therefore, could be extended to other Mg-based intermetallics and beyond (without a reduction requirement). Importantly, the MIMP-dealloying process facilitates the sustainable fabrication of Si with the nanoporous microstructure that is ideal for its extended use in LIBs (as demonstrated by our preliminarily electrochemical results) and potentially useful for a still wider range of applications. By establishing a deeper understanding of the chemistry and physics of the MIMP process, both *ex situ* and *in situ*, it should be possible to optimize these energy-efficient routes toward an expansive inventory of functional nanomaterials.

## IV Experimental Section

### Synthesis

#### SiO_2_ Reduction

One millimole of SiO_2_ powders (quartz, 99.5%, 400 mesh, Alfa Aesar) and 5 mmol of Mg powders (99.8%, 325 mesh, Alfa Aesar) were thoroughly mixed and transferred into a quartz tube (MW-transparent).^48^ Sample preparation was performed entirely inside an N_2_-filled LABstar glovebox (mBRAUN) with H_2_O and O_2_ levels below 0.5 ppm. The apparatus for the MIMP reactions is shown in Figure S1. Initially, the reaction tube was closed by a fitted poly(tetrafluoroethylene) (PTFE) Young’s tap inside the glovebox, transferred outside the glovebox to a single-mode MW cavity reactor (CEM Discovery, 2.45 GHz), and connected to a vacuum line. The first MW irradiation was performed at 200 W for 60 s under a static vacuum of 1.0 × 10^–1^ mbar, after which the tube was cooled naturally. After grinding the product in a fumehood, the second MW irradiation was performed at 200 W for 90 s under a static vacuum of 1.0 × 10^–1^ mbar. The tube was cooled naturally, and the product powders were collected. In separate experiments, we noted that doubling the reactant amount in the second MW irradiation cycle did not require additional irradiation time to increase the yield two-fold. Further scaling up would require modifications to the reaction configuration.

#### Fabrication of NP Si

The MW-obtained Mg_2_Si and MgO powder mixture from the SiO_2_ Reduction section was transferred into an alumina crucible and thermally dealloyed in air by heating at 550 °C for 8 h in a box furnace in a fumehood. After naturally cooling to room temperature, the powders were immersed in a 1 M HCl aqueous solution for 30 min, centrifuged, and washed with deionized water (3 times) and ethanol (3 times). The washed powders were dried in an oven at 60 °C for 3 h. Dry NP Si powders were stored in a N_2_-filled glovebox for further tests and characterization. An amount of 100–103 mg of NP Si powders was consistently obtained from 4 mmol of SiO_2_ precursor (i.e., theoretically containing 112 mg of Si), attesting to the high yield percentage (89.3–92.0%) through the MIMP-dealloying routes, which can be optimized in potential large-scale productions.

#### Synthesis of Mg_2_Si from Si and Mg

This reaction used nearly identical setups as with the SiO_2_–Mg reaction in the SiO_2_ Reduction section, even though the Si (99.8%, 325 mesh, Alfa Aesar) and Mg (99.8%, 325 mesh, Alfa Aesar) powder mixture (Mg/Si in a molar ratio of 2.3:1.0) was put within an alumina crucible (MW-transparent) inside the quartz tube.^48^ The parameters used were 200 W of MW irradiation for 60 s under a static vacuum of *P* < 10^–6^ mbar.^48^

### Electrochemical Measurements

The NP Si powders were mixed with Super P carbon black (99+%, metal basis, Alfa Aesar) and a sodium alginate (Sigma-Aldrich) binder in a weight ratio of 60:20:20 to form a homogeneous slurry. The slurry was coated onto a copper foil (10 μm in thickness) and dried at 80 °C for 12 h under a vacuum of 2.0 × 10^–2^ mbar to fabricate the NP Si electrodes. The mass loadings of active materials are ∼0.4–0.45 mg/cm^2^. Half-cells were assembled using MIT split-able cells with inner diameters of 20 mm; the NP Si electrode (16 mm in diameter) was used as the working electrode, and a piece of glass-fiber D (GF/D, 20 mm in diameter, Whatman) filter paper was used for the separator. Li foil (99.9%, metal basis, 0.75 mm thickness, Alfa Aesar) was manually polished and prepared into a clean Li disk (19 mm diameter) as the counter electrode. The electrolyte was 1.0 M LiPF_6_ in ethylene carbonate/dimethyl carbonate (EC/DMC, 50/50, v/v; Sigma-Aldrich). All half-cells were assembled in an Ar-filled glovebox with the H_2_O and O_2_ contents below 0.5 ppm. (Dis)charging cycles were performed at room temperature using a galvanostatic programmable battery tester (Neware, CT-4008, 5 V 10 mA) at different current densities with a cutoff potential range of 0.05–1.50 V. The EIS measurement was performed after battery-cycling at the open-circuit voltage on a Biologic SP-150 potentiostat in the frequency range of 100 kHz–10 mHz using an amplitude of 10 mV. To perform postcycling characterization of the electrode, the half-cell was disassembled in the glovebox. Then, the electrode was thoroughly washed with DMC (>99.5%, anhydrous, Sigma-Aldrich), dried under vacuum, and stored in the glovebox for further characterization.

Full characterization details are available in Supporting Information 1.

## Supporting Information Available

Supporting Information 1: Documentation of supplementary experimental and analysis details, plus supporting figures and tables (PDF)
Supporting Information 2: Constructed 3D TXM tomograms and accompanying video of a particle of as-synthesized hierarchical nanoporous Si (MP4)
Supporting Video 1: first MW irradiation of Mg/SiO_2_ at 200 W under a static vacuum (*P* = 1.0 × 10^–1^ mbar); movie played at 1x speed with a frame rate of 60 frames per second (MP4)
Supporting Video 2: second MW irradiation of Mg/SiO_2_ at 200 W under a static vacuum (*P* = 1.0 × 10^–1^ mbar); movie played at 1x speed with a frame rate of 60 frames per second (MP4)
