# Supplementary material for: Carbon-Free Conversion of SiO2 to Si via Ultra-Rapid Alloy Formation: Toward the Sustainable Fabrication of Nanoporous Si for Lithium-Ion Batteries
Source: ACS Appl Mater Interfaces. 2023 Jul 19;15(30):36076–85. doi: 10.1021/acsami.3c02197 (PMC10401573; doi:10.1021/acsami.3c02197)
Supplement: Supplementary file 1 — am3c02197_si_001.pdf [file am3c02197_si_001.pdf]

Supporting Information 1 (12 Pages, 7 Figures, 5 Tables)

Carbon-free Conversion of SiO_2 to Si via Ultra-Rapid Alloy Formation: Towards the Sustainable Fabrication of Nanoporous Si for Lithium Ion Batteries

Zhen Fan[1], Wei-Ren Liu[2], Lin Sun[1], Akira Nishio[3], Robert Szczęsny[4], Yan-Gu Lin[5], Shigeto Okada[3], and Duncan H. Gregory[1]*.

1. WestCHEM, School of Chemistry, University of Glasgow, Glasgow, G12 8QQ, United Kingdom.
2. Department of Chemical Engineering, Chung Yuan Christian University, R&D Center for Membrane Technology, Research Center for Circular Economy, 32023, No. 200, Chun Pei Rd., Chung Li District, Taoyuan City 32023, Taiwan.
3. Institute for Materials Chemistry and Engineering, Kyushu University, 6-1, Kasuga-koen, Kasuga 816-8580, Japan.
4. Faculty of Chemistry, Nicolaus Copernicus University in Toruń, ul. Gagarina 7, 87-100 Toruń, Poland.
5. Research Division, National Synchrotron Radiation Research Center, Hsinchu 30076, Taiwan.

* Corresponding Author. Duncan.Gregory@glasgow.ac.uk; Tel: +44-141-330-8128.

Description of Supporting Information Files

1. Documents

Supporting Information 1 (pdf) – Documentation of supplementary experimental & analysis details plus supporting figures and tables.
Supporting Information 2 (mp4) – Constructed 3D TXM tomograms and accompanying video of a particle of as-synthesized hierarchical nanoporous Si.

2. Videos

Supporting Video 1 (mp4) – 1st MW irradiation of Mg/SiO_2 at 200 W under a static vacuum (P = 1.0* 10^{-1} mbar), movie played at 1X speed with a frame rate of 60 f/s;
Supporting Video 2 (mp4) – 2nd MW irradiation of Mg/SiO_2 at 200 W under a static vacuum (P = 1.0* 10^{-1} mbar), movie played at 1X speed with a frame rate of 60 f/s.

Experimental

Materials Characterization. Powder X-ray diffraction (PXD) was performed using a PANalytical X'pert Pro MPD diffractometer in Bragg–Brentano geometry (Cu−Kα1 radiation λ = 1.5406 Å; accelerating voltage of 40 kV; emission current of 40 mA). Typically, PXD patterns were collected at room temperature over a 2θ range of 15−85° with a step size of 0.0334^o for 10 min for the rapid phase-identification/matching of the MW-irradiated and thermally-dealloyed powders. One PXD dataset was collected from 20-120° (2θ) with a step size of 0.0167^o for 50 min in order to perform Rietveld refinement of the structure of the phase-pure NP Si sample. Rietveld refinement was performed using GSAS via the EXPGUI interface,[1] with a previously published Si structure taken as an initial crystallographic model.[2] Crystallographic models of Mg, MgO, Mg_2Si, and SiO_2 were employed for phase identification/matching purposes.[3-6] Visualization of the crystal structure was fulfilled with the VESTA software package.[7]
Scanning electron microscopy (SEM) and energy dispersive X-ray spectroscopy (EDS) were performed using two instruments: a Carl Zeiss Sigma Variable Pressure Analytical SEM and a Hitachi S-4100 microscope equipped with an INCA X-Act detector (Oxford Instruments Analytical, UK). The NP Si sample was coated with either Pt or Au plasma under vacuum to optimize the SEM image quality. For the cycled Si electrode, the SEM specimen was directly transferred into the SEM instrument without coating in order to keep the air-exposure time < 20 s. Transmission electron microscopy (TEM) and selected area electron diffraction (SAED) of NP Si powders were analyzed by a TEM microscope (FEI, G2 F20X-Twin 200 kV, FEG) equipped with an energy-dispersive X-ray spectrometer (EDAX, RTEM model SN9577, 134 eV) with measurements performed in the TEM mode (for bright-field imaging). A dispersion of the sample was prepared in ethanol by ultrasound; 5 μl of the solution was dropped onto a Cu TEM grid and stored at room temperature until the solvent had completely evaporated.
High resolution Si*2p* X-ray photoelectron spectroscopy (XPS) was performed using a K-Alpha Photoelectron Spectrometer (monochromatic Al-Kα, Thermo Scientific) under vacuum. Brunauer – Emmett – Teller (BET) analyses were performed on N_2 adsorption-desorption isotherms measured at 77 K (using a Micromeritics TriStar 3000 analyzer). The adsorption data were further analyzed using the Barrett–Joyner–Halenda (BJH) method. Samples of *ca.* 100 mg were used for the adsorption-desorption measurements.

Calculations of Porosity. The porosity of the NP Si powders was calculated as below:

$$\textit{Porosity (\%)} = \frac{\textit{Cumulated Pore Volume}}{\textit{Cumulated Pore Volume} + \textit{Volume of Si}} * 100\% \qquad \textbf{(S1)}$$

The BJH desorption cumulative volume of pores (between 1.7 – 300.0 nm in diameter) was 0.262711 cm^3 g^{-1}. This volume was employed as the Cumulated Pore Volume in equation **(S1)**. The volume of Si was calculated as 1 g of Si divided by its density. According to **Table S3** and equation **(S1)**, the calculated porosity is 37.97%.

Transmission X-ray Microscopy (TXM) characterization was conducted at the beamline station of Taiwan Light Source (TLS 01B1), National Synchrotron Radiation Center (NSRRC) in Hsinchu, Taiwan.[8] The Ge (111) toroidal focusing mirror provided monochromatic light

with a photon energy of 8 keV. The transmitted beam passed through a zone-plate and a phase ring to generate an image. The phase ring was positioned at the back focal plane of the zone plate which recorded phase-contrast images at the charge-coupled device (CCD) detector. The beam size for sample observation is about 1 mm × 0.4 mm with an average photon flux of 3×10^{11} photon · sec^{-1} · 200 mA^{-1}. The spatial resolution and field of view of TXM is 50 nm and 15 × 15 μm^2, respectively. TXM 2D tomographic images were collected with a camera binning of 512 × 512 in pixels in the duration of 60 s exposure time. The collected TXM images were further processed and analyzed using ImageJ. The Faproma-alignment algorithm was adopted to correct the vertical and rotational motion errors along each projection to improve 3D reconstructed images. In addition, a maximum likelihood estimation reconstruction method was applied on the 3D image reconstruction using 151 sequential projections along a specific azimuth angle rotation (-90 − 90°). The visible 3D tomographic images and video were reconstructed using Amira 3D image processing software.

Estimation of the Energy Consumption of MIMP vs. Conventional Mg_2Si Synthesis on a Lab Scale.

A rough estimate of the lab scale energy consumption can be performed for the MIMP reduction-magnesiation process of SiO_2 to Mg_2Si, based on the prototype bench-top setup described in the Experimental section in the main paper and Figure S1. This therefore initially assumes a yield of 1 mmol Si per experiment. The equation of the MIMP process is given by:

$$SiO_2 + 4\ Mg\ (plasma) \rightarrow Mg_2Si + 2\ MgO \qquad (i)$$

To synthesize *5 g* of Mg_2Si (as one might expect from a conventional single batch solid-state synthesis performed in a ceramic boat, under inert gas in a tube furnace), *i.e.* ~ 65 mmol, the MIMP reaction would require:

Applied power × time × N° of experiments batches

⇒ 0.2 kW × (2.5/60) h × 65 = 0.542 kW h of electricity.

Based on Equation (i), no CO_x gases are chemically emitted during the MIMP process.

The conventional high temperature synthesis process from SiO_2 to Mg_2Si, as summarized in the main paper proceeds *via*:

$$SiO_2 + 2\ C \rightarrow Si + 2CO \qquad (ii)$$

$$2\ Mg + Si \rightarrow Mg_2Si \qquad (iii)$$

Here, Reaction (ii) is a high temperature carbothermal reduction (⩾ 1900 °C) and Reaction (iii) is a high-temperature solid-state synthesis under flowing inert gas (e.g. in a tube furnace). To synthesize 5 g of Mg_2Si, Reaction (ii) would typically utilize a 6 kW electrical induction or arc furnace for 1 h thus consuming 6 kW h of electricity and also emitting ~ 130 mmol of CO (~ 3.7 g). Reaction (iii) could be performed with a tube furnace (3.6 kW) under flowing Ar gas for 10 h, thus consuming 36 kW. The entire process (ii + iii) hence requires approximately 42 kW h of energy (and emits CO gas). (Using commercial Si in reaction (iii) would reduce the energy consumption based on an energy cost of 12 kW h kg^{-1} to produce Si from SiO_2 industrially, as described in Ref 33 in the main paper).

Although the current bench-top setup is only at the prototype stage, the results demonstrate the promise of scaling up the MIMP process. Further scale-up can potentially be achievable through alternative commercial and/or bespoke reactors with larger and/or multiple cavities and optimized designs, on the foundation of which, the energy efficiency of the MIMP physiochemistry will promisingly further increase.

Supporting Figures and Tables

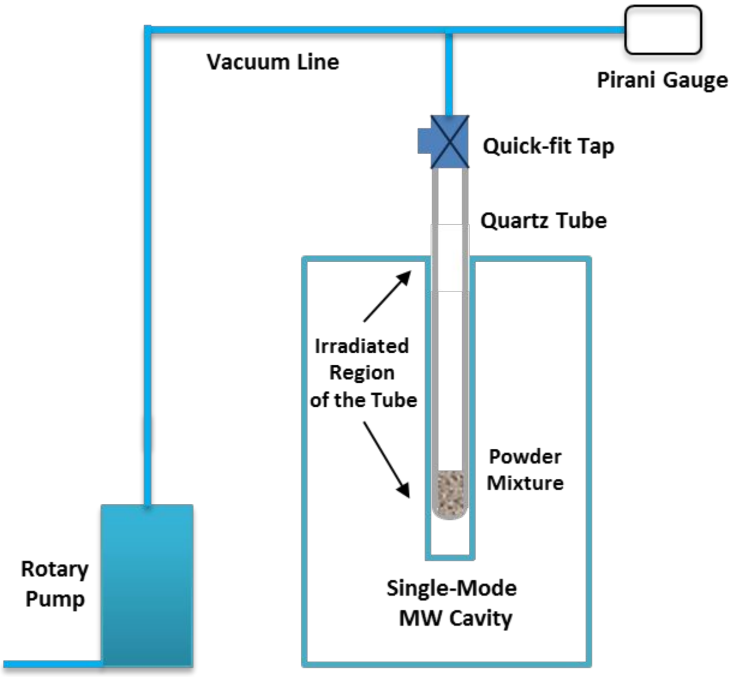

Figure S1. Experimental setup for the MIMP synthesis.

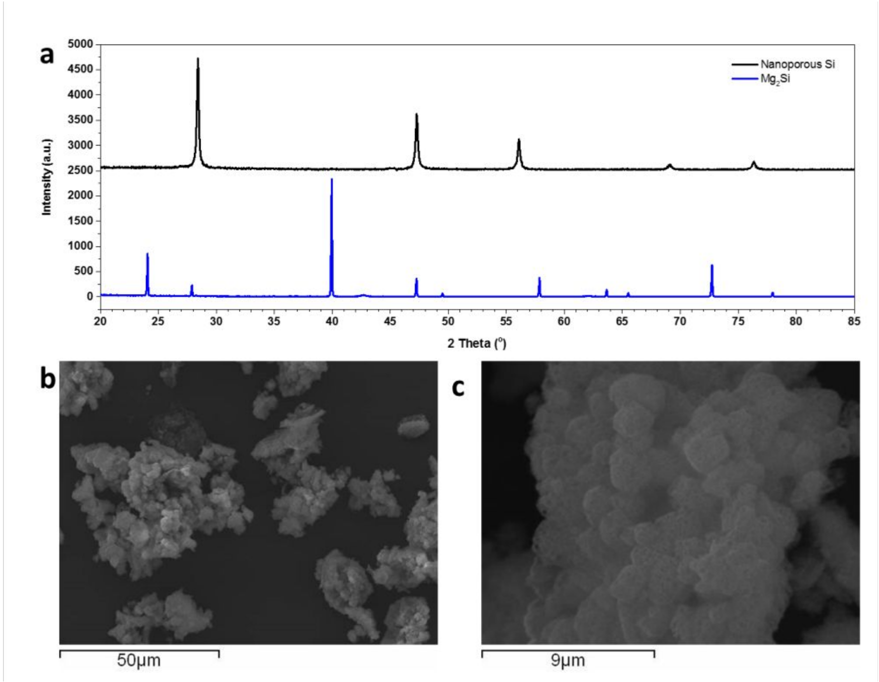

Figure S2. (a) PXRD patterns of MIMP-synthesized Mg_2Si from Mg and Si powder mixture and the corresponding NP Si following the same dealloying, washing, and drying methods as the main paper; and **(b)** low-magnification SEM images of the NP Si in (a).

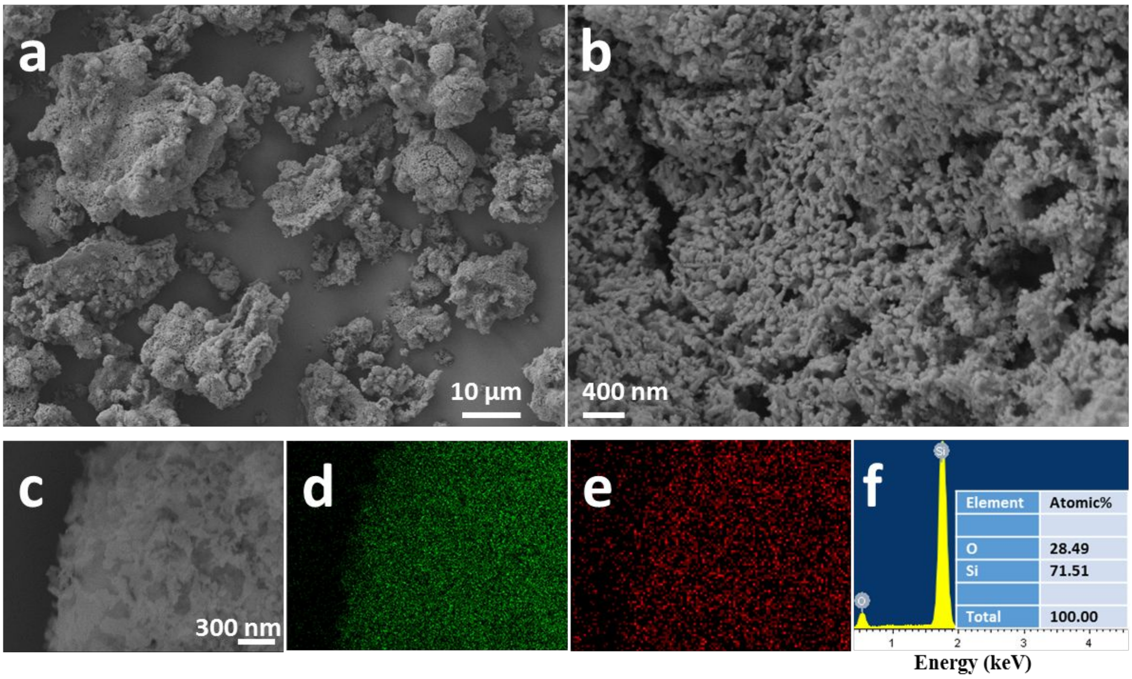

Figure S3. (a-c) Additional SEM images of nanoporous Si sample (in the main paper); **(d, e)** elemental mappings of Si (d) and O (e) in (c); and **(f)** the corresponding areal EDS spectrum.

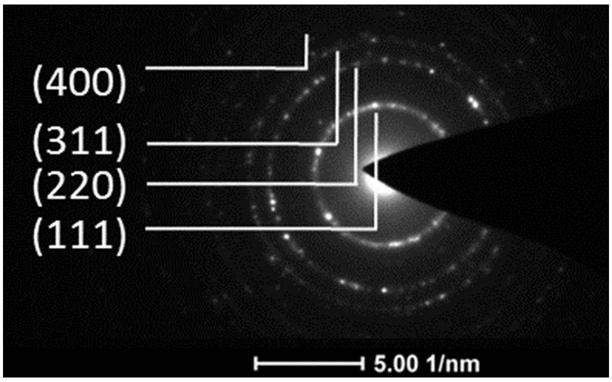

Figure S4. Indexed SAED pattern of NP Si in Figure 3f in the main paper (see also the inset SAED in Figure 3f).

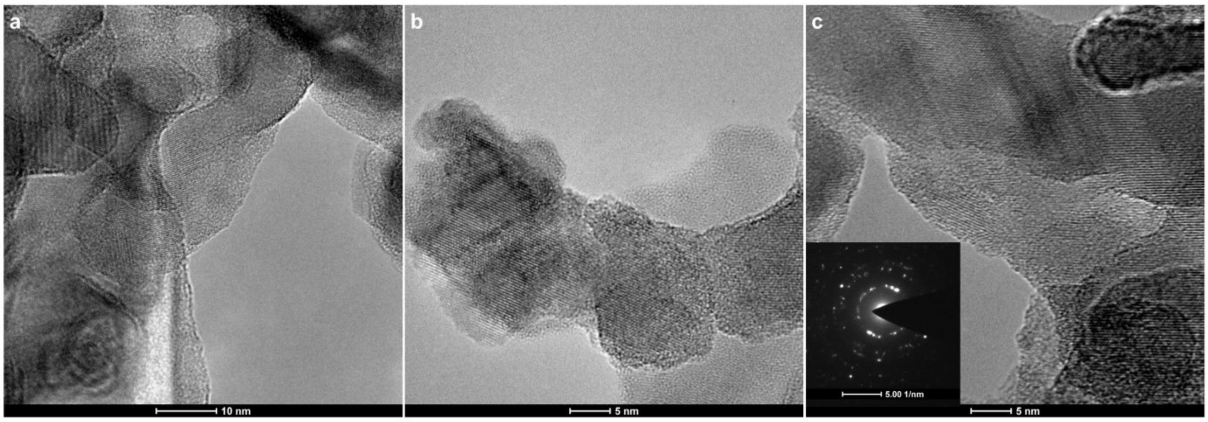

Figure S5. Additional HRTEM images confirming the highly crystalline characteristics of the NP Si products. Inset (c): SAED patterns taken corresponding to (c).

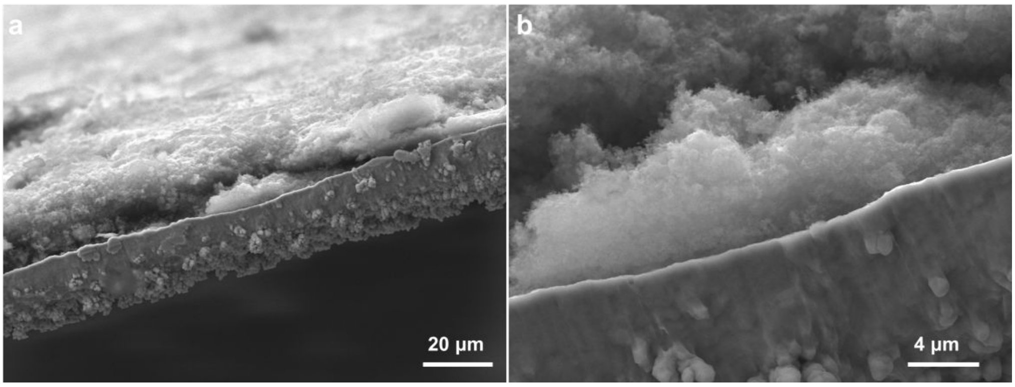

Figure S6. Cross-sectional SEM images of the as-prepared electrodes from NP Si prior to electrochemical cycling (see also Figures 5a,b).

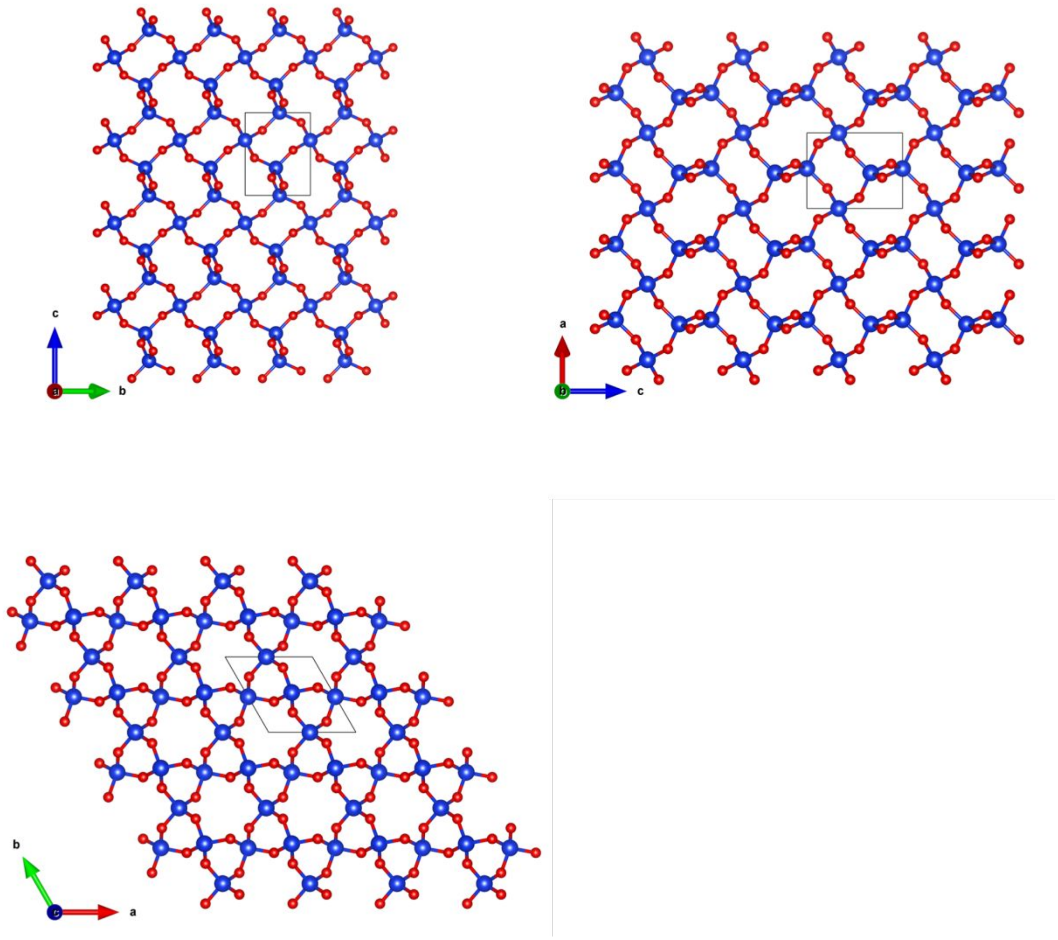

Figure S7. The visualization of frames in the crystal structure of SiO_2 (quartz) (model adopted from [3]). Atoms: red – oxygen, royal blue – Si.

Table S1. Crystallographic data obtained from the Rietveld refinement for the as-synthesized NP Si.

**Chemical Formula**	**Si**
Crystal System	Cubic
Space Group	*F d -3 m* (227)
Lattice Parameter, *a* / Å	5.4316(3)
Formula Weight / g mol^{-1}	224.688
Formula Units, *Z*	8
Calculated Density / g cm^{-3}	2.328
No. of Variables	31
No. of Observations	6282
R_{wp}	0.1302
R_p	0.0979
χ^2	1.277

Table S2. Refined atomic parameters for NP Si.

**Atom**	**Wyckoff Symbol**	***x***	***y***	***z***	**100*U_{iso} / Å^2**	**Occupancy**
Si	8*a*	0.1250	0.1250	0.1250	2.84(5)	1.0

Table S3. Surface area and porosity information from BET Measurements for NP Si.

BET Surface Area (m^2/g)	63.2136
Langmuir Surface Area (m^2/g)	97.9013
BJH Adsorption Cumulative Surface Area of Pore between 1.7 – 300.0 nm diameter (m^2/g)	68.4421
BJH Desorption Cumulative Surface Area of Pore between 1.7 – 300.0 nm diameter (m^2/g)	69.4463
BJH Adsorption Cumulative Volume of Pores between 1.7 – 300.0 nm diameter (cm^3/g)	0.262924
BJH Desorption Cumulative Volume of Pores between 1.7 – 300.0 nm diameter (cm^3/g)	0.262711
Porosity (%)	37.97

Table S4. Profile fitting of the XPS spectrum shown in Figure 3i.

**Peak**	**Peak Position (eV)**	**Atomic %**
Si *2p doublet*	99.31 and 99.78	43.41
Si *2p* (SiO_2)	103.38	56.59

Table S5. Fitted EIS spectrum results in Figure 5f.

**Component**	**Value (ohm)**
R_s	5.092
R_{ct}	26.17
CPE	*CPE-T*: 4.05*E-05
	CPE-P: 0.68
Z_w	Z_w-*R*: 33.82
	Z_w-*T*: 0.006
	Z_w-*P*: 0.27

References

[1] Toby, B.H. EXPGUI, a Graphical User Interface for GSAS. *J. Appl. Cryst.* **2001**, 34(2), 210-213.

[2] Többens, D. M.; Stüßer, N.; Knorr, K.; Mayer, H. M.; Lampert, G. E9: the new high-resolution neutron powder diffractometer at the Berlin neutron scattering center [C]. *Materials Science Forum. Trans Tech Publications Ltd.*, **2001**, 378, 288-293.

[3] d'Amour, H.; Denner, W.; Schulz, H. E. I. N. Z. Structure de-termination of α-quartz up to 68 x 108 Pa. *Acta Crystallographica Section B: Structural Crystallography and Crystal Chemistry*, **1979**, 35(3), 550-555.

[4] Saravanan, R.; Robert, M. C. Local structure of the thermoelectric material Mg_2Si using XRD. *J. Alloy Compd.*, **2009**, 479(1-2), 26-31.

[5] SASAKI, S.; FUJINO, K.; TAKÉUCHI, Y. X-ray determination of electron-density distributions in oxides, MgO, MnO, CoO, and NiO, and atomic scattering factors of their constituent atoms. *P. JPN Acad. B-Phys.*, **1979**, 55(2), 43-48.

[6] Owen, E. A.; Pickup, L.; Roberts, I. O. Lattice constants of five elements possessing hexagonal structure. *Zeitschrift für Kristal-lographie-Crystalline Materials*, **1935**, 91(1-6), 70-76.

[7] Momma, K.; Izumi, F.VESTA3 for Three-Dimensional Visualization of Crystal, Volumetric and Morphology Data. *J. Appl. Cryst.*, **2011**, 44, 1272-1276.

[8] Hsieh, C.C.; Lin, Y.G.; Chiang, C. L.; Liu, W.R. Carbon-coated porous Si/C composite anode materials via two-step etching/coating processes for lithium-ion batteries. *Ceram. Int.*, **2020**, *46*(17), 26598-26607.
